# 电场辅助分子印迹技术进展

**DOI:** 10.3724/SP.J.1123.2025.05006

**Published:** 2026-01-08

**Authors:** Jiangyi WU, Xiaojia HUANG

**Affiliations:** 1. 厦门大学嘉庚学院，环境科学与工程学院，福建 漳州 363123; 1. School of Environmental Science & Engineering，Xiamen University Tan Kah Kee College，Zhangzhou 363123，China; 2. 厦门大学环境与生态学院，滨海湿地生态系统教育部重点实验室，福建 厦门 361005; 2. Key Laboratory of the Ministry of Education for Costal and Wetland Ecosystem，College of the Environmental and Ecology，Xiamen University，Xiamen 361005，China

**Keywords:** 分子印迹技术, 电场, 分子印迹聚合物, 样品前处理, 综述, molecular imprinting technology （MIT）, electric field, molecularly imprinted polymers （MIPs）, sample pretreatment, review

## Abstract

分子印迹技术（molecular imprinting technology， MIT）借鉴抗体-抗原特异性识别机制，能够高度精准地对目标物质进行选择性萃取，在分离、检测等领域极具应用潜力。但传统MIT在材料制备、样品前处理及检测分析中存在诸多亟待解决的问题：制备的分子印迹聚合物（molecularly imprinted polymers，MIPs）存在印迹位点不均一、模板分子残留严重、机械性能差等缺陷；以MIPs为吸附剂的前处理方法因目标物选择吸附速率慢而耗时，且特异性识别性能有待提升；基于MIPs的检测手段灵敏度低，检测耗时长，难以现场实时监测。这些问题制约了MIT的发展与广泛应用。近些年，电场辅助技术与MIT结合为解决上述问题提供了有效策略。制备MIPs时，在聚合体系中引入电场，使带电模板分子与功能单体受电场力定向移动，促使单体更有序地围绕模板分子排列，从而制得印迹位点均匀、分子取向性良好的MIPs。在样品前处理过程中，外部电场所提供的电泳驱动力可提升MIPs对目标物的传质速率，缩短吸附与解吸时间，优化其特异性识别性能。此外，MIPs电化学传感器的发展及其与微流控技术的结合显著提升了MIPs在检测领域的实用性。本文重点阐述电场在MIPs制备、样品前处理及检测分析三大关键环节的具体应用与作用机制，总结电场辅助MIT在环境监测、生物医学、食品安全等领域的应用前景，并展望了未来发展方向。

## Abstract (en)

Molecular imprinting technology （MIT） simulates the specific recognition mechanism between antibodies and antigens， and can construct separation media with specific recognition performance， demonstrating unique advantages in the fields of separation， purification， and trace detection in complex sample matrices. Molecularly imprinted polymers （MIPs）， the core of MIT， form three-dimensional imprinting cavities matching the spatial structure and chemical properties of the target substance via three key steps： template self-assembly with functional monomer， cross-linking polymerization， and template elution. The prepared MIPs present highly selective extraction and recognition of the target substance. However， traditional MIT faces critical challenges in material preparation and application. During MIPs synthesis， due to the randomness of polymerization reactions， the resulting MIPs often have uneven distribution of imprinting sites and lack molecular orientation. This not only reduces the mechanical performance of MIPs， but also affects their specific recognition performance. In addition， incomplete removal of template molecules severely reduces the number of effective imprinting sites and may interfere with the accuracy of subsequent detection results. In the pre-treatment application of MIPs adsorbents， the mass transfer rate of the target substance in the sample solution is slow， which prolongs the adsorption equilibrium time of MIPs on it. In the sensor detection based on MIPs， there are inherent issues such as fluorescence quenching and insufficient signal amplification， which further limit the detection sensitivity. These challenges significantly limit the application of MIT in fields like environmental monitoring， biomedical diagnostics， and food safety analysis. In recent years， the cross fusion of electric field assisted technology and MIT has provided innovative strategies to solve the above-mentioned problems. During MIPs preparation， an external electric field can be applied to provide electrophoretic driving force to assist in regulating the directional self-assembly of functional monomers and template molecules. This can promote the ordered arrangement of imprinting sites at the micro level of MIPs prepared， achieving material uniformity of MIPs. For template molecule elution， applying an electric field can provide electrostatic repulsion between MIPs and template molecules. This can effectively weaken the inter-molecular forces between MIPs and template molecules， facilitate the diffusion of template molecules into the desorption solution， reduce the residual template molecules， and avoid the detection of “false positives”. During the sample pretreatment process， electrophoretic force significantly enhances the mass transfer rate of the target substance， prompting it to aggregate around the MIPs adsorbent and achieve the purpose of pre-concentration. This can not only shorten the adsorption equilibrium time of MIPs for target substances， achieve rapid enrichment， but also effectively drive away interfering substances with opposite electrical properties， improving the selective extraction performance of MIPs for target substances. In the detection and analysis stages， the signal enhancement effect induced by the electric field can significantly improve the response sensitivity of the sensor. Combining with electrochemical and fluorescence detection methods， ultra sensitive detection of the target substance can be achieved. Moreover， MIP-based sensors can be integrated with microfluidic technology to achieve rapid detection and analysis of trace samples， greatly expanding the practicality of MIPs in on-site detection and analysis applications. The present review systematically elaborates the mechanism of electric field in the three key links of MIPs preparation， sample pretreatment and detection analysis. At the same time， it summarizes the application prospects of electric field assisted MIT in environmental monitoring， biomedical， food safety and other fields. In addition， the future development direction of electric field assisted MITs is proposed.

随着人们对环境健康、食品安全等问题的日益关注，对各类复杂基质的样品前处理技术随之飞速发展。目前，开发高萃取性能吸附材料是样品前处理技术的研究热点，已有诸多技术用于研制各种萃取介质^［1，2］^。其中，Polyakov^［3］^于1931年首次提出的基于模拟抗体和抗原之间识别机制的分子印迹技术（molecular imprinting technology，MIT）引起人们的广泛关注。随后，1972年，Wulff等^［4］^通过“共价印迹法”成功制备了分子印迹聚合物（molecularly imprinted polymers，MIPs），将MIT从理论转化为实际应用。但是，模板分子与材料间的共价键结合力太强，导致制备出的材料难以实现对模板分子完全去除，限制了MIT的应用。1993年，Mosbach等^［5］^以“非共价印迹法”合成MIPs，极大地推动了MIT的研究进程。作为一种新兴的仿生学工艺，MIT通过模拟抗原-抗体间的特异性识别性能及专一高亲和力特点，实现了对复杂样品中痕量目标物质的选择性结合^［6］^。同时，凭借所具有的独特的分子特异性识别能力，MIT在分离、检测等领域有着广泛的应用前景^［7-9］^。然而，传统MIT在材料制备、样品前处理和检测分析等方面仍存在一些局限性。经传统MIT制得的MIPs材料存在印迹位点不均一、模板分子残留严重以及材料本身机械性能较差等问题；在利用MIPs进行样品前处理时，往往存在萃取过程较慢、印迹效果不明显和适用性较差等问题；而在利用MIPs进行检测方面，存在检测灵敏度较低、检测时间较长和现场实时监测困难等问题^［10-13］^。

近年来，借助电场辅助技术，MIT出现了新的发展机遇，电场的引入，从多个关键环节显著改善和拓展了MIT的应用。在制备MIPs时，通过在聚合体系中引入电场，使带电的模板分子和功能单体受电场力作用定向移动，促使功能单体更有序地围绕模板分子排列，从而制备出具有良好分子取向性的MIPs^［14-19］^，这不仅提高了MIPs的特异性识别性能，还因其聚合排列有序，所制得的MIPs具有更好的机械稳定性能^［20-23］^。此外，在洗脱模板分子时施加电场，模板分子受到电泳驱动力作用，更易从印迹腔中脱离出来，可避免“假阳性”现象^［18］^。利用MIPs进行样品前处理时，在吸附过程施加电场，诱导两电极之间产生电势差，在电泳驱动力作用下，样品中带电荷的目标物质会朝着带相反电荷的电极迁移，克服了传统自由扩散效率低的限制，从而达到促进吸附和浓缩的目的^［24-29］^。电泳驱动力能够增强目标物质与印迹位点之间的结合力，从而抑制对干扰物质的吸附，可有效改善MIPs的特异性识别性能^［17，30-34］^。此外，电场辅助下，MIPs可以通过与微流控等技术结合，在样品前处理中实现在线分离与富集，可拓展适用范围^［35-37］^。在分析检测方面，电场能够增强MIPs与目标物质结合后的检测信号。在电化学检测中，电场作用下目标物质与MIPs结合后，会引起材料表面电子转移速率的变化，从而放大电流、电位等电化学信号，实现对低浓度目标物质的高灵敏度检测^［38-43］^。在荧光检测中，电场可调节MIPs与荧光标记物的相互作用，增强荧光信号，提高检测的灵敏度^［44，45］^。另外，研究表明，基于电场的分子印迹传感器，不仅实现了对目标物质的快速检测，而且可以实时监测目标分子的浓度变化^［19，33，36，46，47］^。

基于电场辅助MIT的发展，本文将对目前相关研究工作进行概述，依次对电场在MIPs制备过程、样品前处理过程和检测分析中的应用进行总结及评述，同时就电场辅助MIT今后发展的趋势进行展望。

## 1 电场在MIPs制备过程中的作用

传统MIPs的制备方法多样，常用的主要包括本体聚合法^［48，49］^、悬浮聚合法^［50-52］^、沉淀聚合法^［53，54］^和溶胶-凝胶聚合法^［55，56］^。这些制备方法虽各有特色，但原理大致相同，均以聚合反应完成制备。然而，制备过程均需综合考虑聚合溶液中各组分间（模板分子、功能单体、交联剂、致孔剂和引发剂）的比例、聚合反应条件和模板分子去除方法等因素，以制备出具有更好特异性识别性能的MIPs材料^［57，58］^。虽然MIPs的制备方法多样且发展较为成熟，但亦存在影响MIPs印迹效果和使用寿命的印迹位点不均一、模板分子残留严重以及材料本身机械性能较差等问题^［59-61］^。然而，在制备MIPs过程中引入电场，有利于材料在空间结构上的有序组装以及后续对模板分子的有效去除。同时，外加电场能够加快聚合反应进程，有效缩短制备时间。

### 1.1 电场驱动MIPs聚合

在MIPs制备过程中，聚合反应是关键步骤之一。在不加电场的聚合反应中，模板分子、功能单体和交联剂均匀分散于致孔剂中，在引发作用下自行完成局部聚合，随后再相互交联于一体。该过程随着反应时间的延长，MIPs的交联度随之提升^［62］^。而电场的引入可以促进模板分子、功能单体、交联剂等分子的迁移和扩散，使它们更快速地到达反应位点，从而加速聚合反应的进程^［63，64］^。这不仅能缩短反应时间，还可获得具有良好结构和性能的MIPs。Huang等^［65］^以2，4，6-三溴苯酚为模板分子，结合MIT和壳聚糖聚合物通过电沉积手段在金电极表面制备MIP材料。该材料制备过程简单快捷，仅需将预处理过的金电极浸泡于聚合溶液中，在-1.0~1.0 V范围内以100 mV/s的扫描速率重复30次，利用电位动力学循环获得沉积物，去离子水冲洗干燥后即可制得MIP功能电极。Prasad等^［66］^以电聚合技术在多壁碳纳米管改性的铅笔石墨电极上制备了MIPs材料，用于蛋氨酸对映异构体的特异性识别检测。在电聚合过程中，蛋氨酸瞬间被氧化成蛋氨酸砜作为模板分子，利用静电驱动作用加速模板加合物中的氢键连接，从而缩短聚合反应时间。

### 1.2 电场调控MIPs空间结构

模板分子和功能单体的自组装情况对MIPs的特异性识别性能至关重要。在传统MIPs制备过程中，聚合溶液中的模板分子、功能单体和交联剂在没有外加驱动力的作用下有序自组装情况较差，以至于制备得到的MIPs在微观空间结构上印迹位点不均一。然而，外加电场对MIPs的结构和形态具有良好的调控作用。电场能够提供一定的电泳驱动力，促使带电的模板分子和功能单体发生定向移动和有序排列^［67-69］^。通过改变电场的参数，如电场强度、方向和持续作用时间等，可以影响聚合物的生长方式和聚集状态，从而制备出具有特定孔结构和形态的MIPs^［70］^。An等^［71］^利用电沉积技术在玻碳电极表面制备了MIP材料，用于对牛奶样品中三聚氰胺的灵敏检测。在制备过程中，研究优化了电沉积制备MIP的循环扫描次数以及扫描速率。实验结果表明，循环电沉积次数过多会导致MIP涂层太厚不利于模板分子的洗脱，而扫描速率过高则会影响MIP涂层的密度从而影响印迹位点的产生。此外，在电场作用下所制得的MIPs空间结构排列更加有序，形成的孔道更加规整，所含的印迹空腔也具备良好的分子取向性，有利于提高MIPs对目标物质的识别能力和吸附性能。Huang等^［17］^在预处理过的不锈钢丝表面通电制备了一种可选择性萃取植物激素的MIP微电极，并将其用作电场辅助固相微萃取技术中的萃取介质，在最佳条件下与高效液相色谱-二极管阵列检测器（HPLC-DAD）联用，实现了对环境水样和农产品中痕量植物激素的高灵敏度检测。如图1所示，该微电极的制备过程中全程施加外部电场，确保制得的MIP含有的印迹位点具有一定的分子取向性，以便后续电场辅助固相微萃取时样品溶液中目标物质能够定向移动顺利与印迹空腔结合。在施加电场下，MIP微电极对目标物的印迹因子（imprinting factor，IF）为2.47~3.13，而不施加电场，所制得的MIP微电极的IF值则只有1.50~2.09。

**图1:**
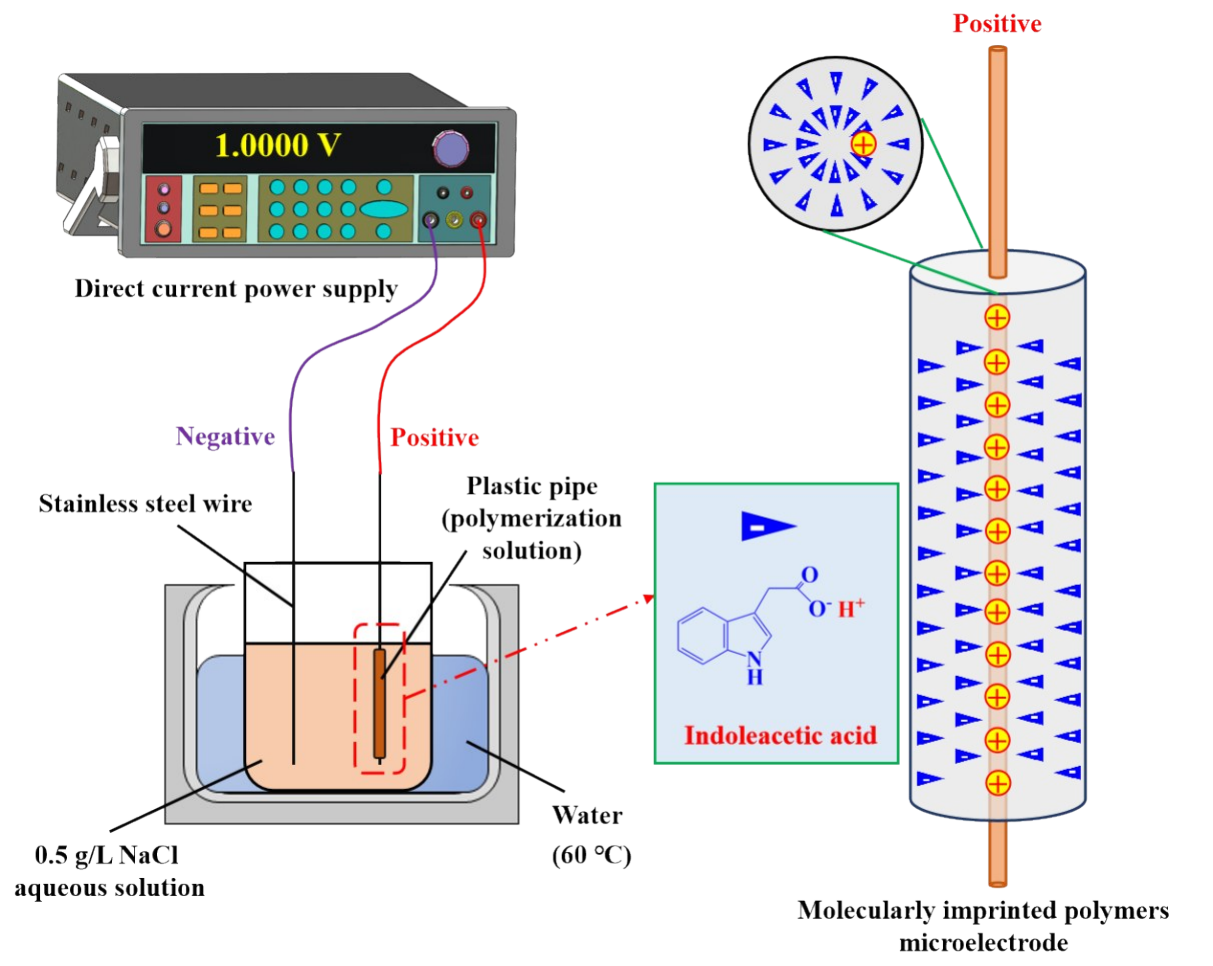
电场辅助MIP微电极的制备示意图

### 1.3 电场辅助MIPs原位制备

利用电场可以在特定的支撑体（金属丝、金属片等）或电极表面实现MIPs的原位制备，这种通电原位制备方法可以使MIPs与支撑体更好结合，形成具有特定功能的吸附电极或传感器^［72-75］^。通电原位制备方法相对传统涂敷制备方法具有较大的优势。一方面，经通电原位制得的MIPs与支撑体之间的结合更加牢固，避免了传统方法中可能出现的MIPs与支撑体分离而脱落的问题，提高了吸附电极或传感器的稳定性和使用寿命。另一方面，通电原位制备方法可以精确控制MIPs涂层的厚度和空间结构，根据应用需求调整电场强度、聚合时间等参数，可实现对MIPs性能的优化。Yang等^［76］^利用石墨烯-多壁碳纳米管复合材料对玻碳电极进行改性处理，随后将其浸入含有吡咯和芦丁的水溶液中进行循环电聚合，使MIP材料原位聚合于改性的玻碳电极表面上。该研究还通过电化学阻抗谱表征了该功能电极的界面特性。结果显示，在MIP材料聚合之前，改性电极具有较好的导电性，当电极负载MIP材料后，其导电性随之下降；而去除模板分子后，该电极的导电性能有所回升，这证实了MIP材料成功负载到玻碳电极表面上。Corman等^［77］^利用电聚合技术在玻碳电极表面原位聚合了MIP薄膜，并将其作为电化学传感器，用于血清中特利氟米特的快速检测。为了得到合适的MIP薄膜厚度，研究优化了电聚合循环次数，结果显示循环7次制得的MIP薄膜具有最佳的印迹性能。此外，原位制备过程相对简单，无需复杂的后处理步骤，缩短了制备周期并降低了合成成本。

### 1.4 电场清洗印迹空腔

在MIPs制备完成后，需要将模板分子从聚合物中去除干净，以形成具有特异性识别位点的空穴。常规去除MIPs中模板分子的方法多为溶剂洗脱法，这需要根据模板分子和聚合物的性质，选择合适的溶剂，并通过多次洗脱来完成去除过程^［78-80］^，因此，这一方法虽能实现模板分子的有效去除，但由于依赖反复萃取操作，不仅耗时较长，而且有机溶剂的大量使用也带来了较高的成本与潜在的环境污染风险，在绿色化学和高效生产的背景下，其局限性日益凸显。然而，基于模板分子的带电性，在去除过程中施加与模板分子相反电性的电场。在电场下，模板分子会受到斥力作用，加速其在聚合物中的逆向扩散和溶解进程，从而实现快速洗脱。相较于传统溶剂萃取法，电场辅助洗脱技术大大缩短了MIPs后处理的时间，同时大幅减少了有机溶剂的用量，有效降低了生产成本和环境负担，为MIPs的制备开辟了一条绿色、高效的新路径。

## 2 电场在基于MIPs样品前处理中的作用

传统的样品前处理技术可分为基于液相吸附剂的萃取（solvent-based extraction，SBE）^［81-83］^与基于固相吸附剂的萃取（adsorbent-based extraction，ABE）^［84-86］^。与SBE相比，ABE由于种类多样、萃取性能好和有机溶剂用量少等优点而受到人们的青睐。经过多年的发展，出现了多种形式的ABE，主要包括固相萃取（solid phase extraction，SPE）^［87，88］^、固相微萃取（solid phase microextraction，SPME）^［89，90］^、搅拌棒吸附萃取（stir bar sorptive extraction，SBSE）^［91，92］^、分散固相萃取（dispersive solid phase extraction，d-SPE）^［93，94］^和磁性固相萃取（magnetic solid phase extraction，MSPE）^［95，96］^等。这些萃取模式各具特色，已在环境监测、食品分析和药物分离等领域得到广泛使用。但对于复杂的实际样品，大量存在的干扰物质对目标物的测定存在明显干扰，而MIPs的良好特异性识别能力可以较好地增强样品前处理过程中的抗干扰能力^［97，98］^。然而，基于MIPs的样品前处理技术仍存在因目标物质传质速度慢而导致的萃取效率较低、因MIPs与目标物质结合力较强而导致的洗脱过程缓慢甚至洗脱不完全等问题^［99，100］^。然而，电场辅助技术与MIPs相结合则可以克服上述问题。此外，萃取过程中，电场的施加还可改善MIPs的特异性识别性能，从而增强其对复杂样品基底的抗干扰能力。

### 2.1 电场加速MIPs对目标物质的吸附进程

在传统样品前处理的萃取阶段，MIPs对目标物质的吸附往往受到扩散速率的限制，需经历较长的时间才能达到吸附平衡状态。而施加电场能够提供电泳驱动力，提高目标物的传质速度，促使目标物质快速向MIPs迁移，达到预浓缩的目的^［101-104］^。电场的引入不仅缩短了MIPs对目标物质的吸附时间，还提高了吸附效率，使得MIPs能够更快速地从复杂样品中实现目标物质的分离和富集。Sarkaya等^［105］^以L-组氨酸为模板分子，以*N*-甲基丙烯酰-苯丙氨酸甲酯为功能单体，在硅烷化后的毛细管内聚合制备了整体萃取柱，分别在电压为5、10和15 kV下分离L-组氨酸和D-组氨酸，结果显示，电压越大，异构体分离的速度越快。本课题组^［106］^曾以香草酸为模板分子，利用1-烯丙基-3-甲基咪唑啉（三氟甲基）磺酰基酰亚胺和*N，N，N*-三甲基-1-（4-乙烯基苯基）甲胺氯化物为混合离子液体作为双功能单体，在不锈钢丝表面电聚合制备出MIP吸附剂并用作电场辅助固相微萃取微电极，实现了对环境水样和果汁样品中酚酸类药物的选择性萃取。在该研究中，进行了外加电场与否的对比实验。对照实验结果表明，在吸附时施加0.8 V的电场能够有效促使MIP微电极对酚酸类药物的吸附于55 min达到吸附平衡，而对于未施加电场的对照组，在70 min时间仍未达到吸附平衡。同时，施加电场后，MIP微电极对酚酸类药物萃取效率相对不施加电场时提升了45.9%~62.8%。

### 2.2 电场提高MIPs的特异性识别效果

MIPs在样品前处理过程主要通过自身与目标物质形成共价键或多种分子间作用力完成吸附，且需要目标分子的空间位置与印迹空腔相匹配，该过程存在一定的随机性^［107，108］^，这限制了MIPs的特异性识别性能。然而，外加电场可以有效改善MIPs对目标物质的特异性识别能力^［109-111］^。当样品中存在干扰物质时，目标物质在电场作用下会快速与MIPs上匹配的印迹位点相结合，而干扰物质则难以与这些位点有效结合，并且外加电场能够阻止带有相反电性的干扰物质向吸附位点移动，从而降低对目标物质吸附的干扰。通过调整电场的参数（如电场强度、方向），可以优化MIPs对目标物质的选择性萃取性能，使得目标物质与干扰物质得到更为有效的分离。值得一提的是，通过电场辅助制备得到的MIPs在电场辅助下完成样品前处理时其特异性识别性能将得到更为明显的提升，这主要是因为经电场辅助制得的MIPs具有一定分子取向的印迹空腔^［106］^。吸附过程中，在外加电场辅助下，目标物质会随电泳驱动力移动，并保持一定的分子空间方向性，因此，在遇到方向相同且具有特异性识别性能的印迹空腔时，会在特异性作用力下被迅速吸附。Huang等^［17］^对所制得的MIP功能电极在同一萃取条件下完成通电与不通电对照组的印迹效果对比，结果显示在不通电下，其IF值为2.24~2.60，而通电条件下（1.6 V）其IF值则提升为2.88~3.51，说明外加电场的施加能够有效提升MIP吸附剂对目标物质的特异性识别性能，从而增强其抗干扰能力。

### 2.3 电场辅助在线分离与富集

以MIPs为吸附剂的前处理技术需经过一系列前处理过程完成浓缩和净化后再进行仪器分析检测，样品前处理与仪器检测通常为离线进行，操作较为烦琐。然而，结合微流控等技术，施加外部电场可使MIPs在样品前处理中实现在线分离与富集^［112-115］^。在微流控芯片中集成MIPs和电极，当样品溶液在电场作用下通过含有MIPs的通道时，目标物质被选择性吸附，而其他干扰物质则会随溶液流出。随后，通过改变电场方向或强度大小，可以将吸附在MIPs上的目标物质洗脱下来，直接进入后续的分析检测装置，实现样品前处理与分析检测的在线联用，减少了样品处理步骤和时间，提高了整个分析流程的自动化程度和效率。Wagner等^［113］^以2，4-二氯苯氧乙酸为模板分子，以甲基丙烯酰胺为功能单体，制备出MIP颗粒。随后在10 mm石英池中配制成悬浮溶液搭建模块化微流控系统，结合荧光强度测定，实现了环境水样中2，4-二氯苯氧乙酸的现场监测。Babamiri等^［116］^以胍丁胺为模板分子，以伏安法在导电丝网印刷电极表面制备了一种复合型MIP材料，用作电化学分析传感器。如图2所示，制备好的MIP传感器结合微流控技术，实现了对血浆样品中胍丁胺的快速分离检测。

**图2:**
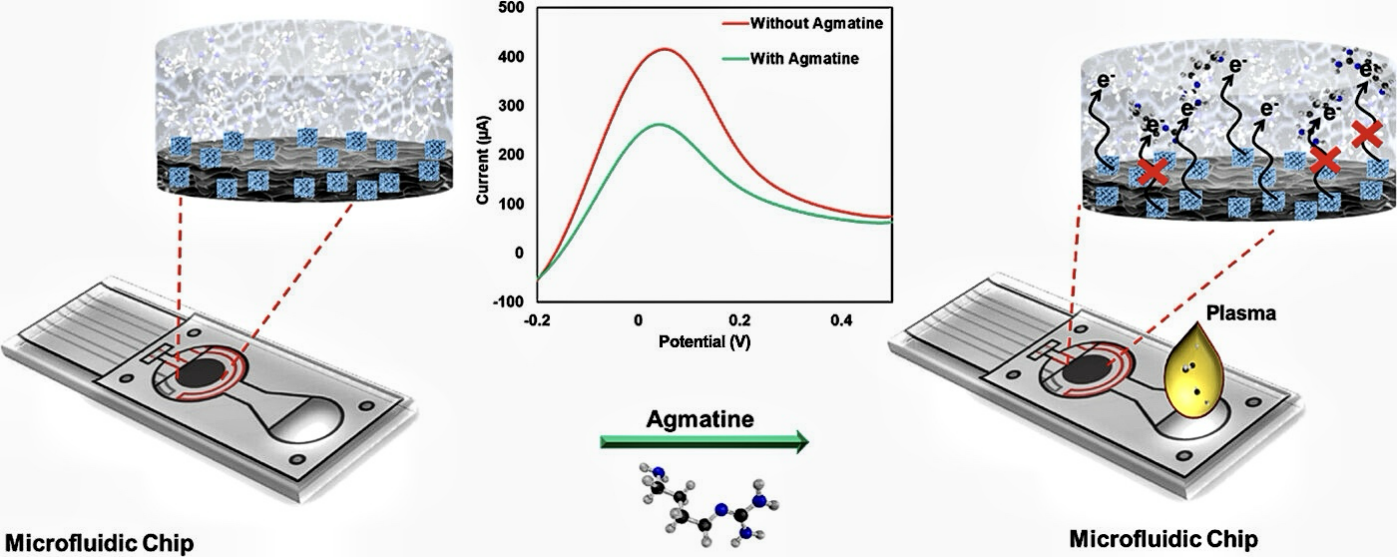
用于检测胍丁胺的MIP生物传感器的示意图^［116］^

### 2.4 电场驱动MIPs洗脱程序

基于MIPs的吸附剂在完成对目标物的特异性吸附后，还需利用合适的解吸溶剂将目标物从MIPs中洗脱转移至溶剂中，便于后续的仪器分析检测。对于一些与MIPs结合紧密的目标物质，需要较长时间来完成解吸过程，这不利于快速检测^［117-119］^。然而，通过对吸附了目标物质的MIPs材料施加相反方向的电场，为MIPs与目标物质之间提供静电斥力，可使目标物质快速从MIPs材料中被释放，从而缩短洗脱过程。本课题组^［106］^详细考察了电场辅助固相微萃取在解吸阶段外加电场强度对于解吸效果的影响，通过在解吸过程中对MIP电极施加与吸附时相反方向的电场，可有效缩短样品前处理过程的解吸时间，同时有利于被吸附的目标物质全部从吸附剂中解吸，避免残留效应。

## 3 电场在检测分析中的作用

凭借独特的分子特异性识别性能，目前MIT也被用于分析检测领域。迄今为止，基于MIT的检测方法主要为电化学检测法和荧光检测法，它们各具特色并在实际应用过程中展现出令人满意的效果^［120-123］^。在电化学检测中，当目标物质与MIPs结合后，会引起材料表面的电子传递过程发生变化，进而引起电极表面电阻、电容等电化学参数的改变^［120，124-126］^。通过电化学工作站等设备对微小改变进行精确测量，可实现对目标物质的定量分析。而在荧光检测中，主要利用目标物质与MIPs的结合影响材料表面的荧光特性，如荧光强度的增强或减弱、荧光发射波长的位移等，通过对这些荧光性质变化的监测，能够快速、准确地检测到目标物质的浓度^［127-129］^。这种基于目标物质与MIPs结合改变材料表面性质的检测机制，为复杂样品中痕量目标物质的测定提供了高效、灵敏、可靠的手段，在环境监测、食品安全、生物医学等众多领域都有着广阔的应用前景^［130-132］^。最近，在检测过程中引入电场，可实现对目标物的增强传质与吸附效率，有利于放大检测信号、提高分析灵敏度。

### 3.1 电场提升MIPs传感器检测速度

在MIPs传感器检测体系中，电场辅助作用与样品前处理阶段的功能类似，其核心机制在于为目标物质提供电泳驱动力。传统的MIPs传感器检测过程中，目标物质在样品溶液中的迁移主要依赖于分子的自由扩散，这种被动传输方式效率较低，导致检测响应时间较长，极大地限制了检测效率的提升。而引入电场辅助后，MIPs传感器的检测性能得到显著优化。当在检测体系中施加合适强度的电场时，带电目标物质会在电场力的作用下实现定向迁移，迁移速率较常规扩散方式大幅提升。这种定向迁移能够促使目标物质迅速突破传质阻力，快速汇聚至MIPs材料表面，并与材料中预先构建的特异性识别位点精准结合。相较于无电场辅助的检测模式，该方法有效缩短了目标物质与MIPs材料的结合时间，使检测响应速度得到显著提高。电场辅助MIPs传感器检测技术不仅为快速、高效的物质检测提供了新途径，还在环境监测、生物分析、食品安全等对检测时效性要求极高的领域，展现出广阔的应用前景和巨大的发展潜力^［133］^。

### 3.2 电化学信号放大

外加电场能够有效助力MIPs材料在检测时的信号放大。如图3所示，基于电化学检测的分子印迹传感器，当目标物质与MIPs材料结合后，会引起材料表面电化学性质的变化，并且通过施加电场，可以进一步增强这种变化，从而实现信号放大^［134，135］^。此外，电场还可以与荧光检测相结合，通过电场调控荧光分子与MIPs材料的相互作用，实现荧光信号的放大和检测。Rao等^［38］^以三聚氰胺为模板分子，以甲基丙烯酸为功能单体，利用电沉积技术在处理过的玻碳电极表面制备MIP涂层。该研究通过对该传感器施加不同大小的电流来考察其对三聚氰胺的检测灵敏度，结果表明电流的施加能够有效提升电极表面的电催化效果和电化学传感器的灵敏度，检出限（limit of detection， LOD）可达1.39×10^-6^ μmol/L。表1总结了目前外加电场下，MIPs传感器电化学检测分析的一些应用实例。

**图3:**
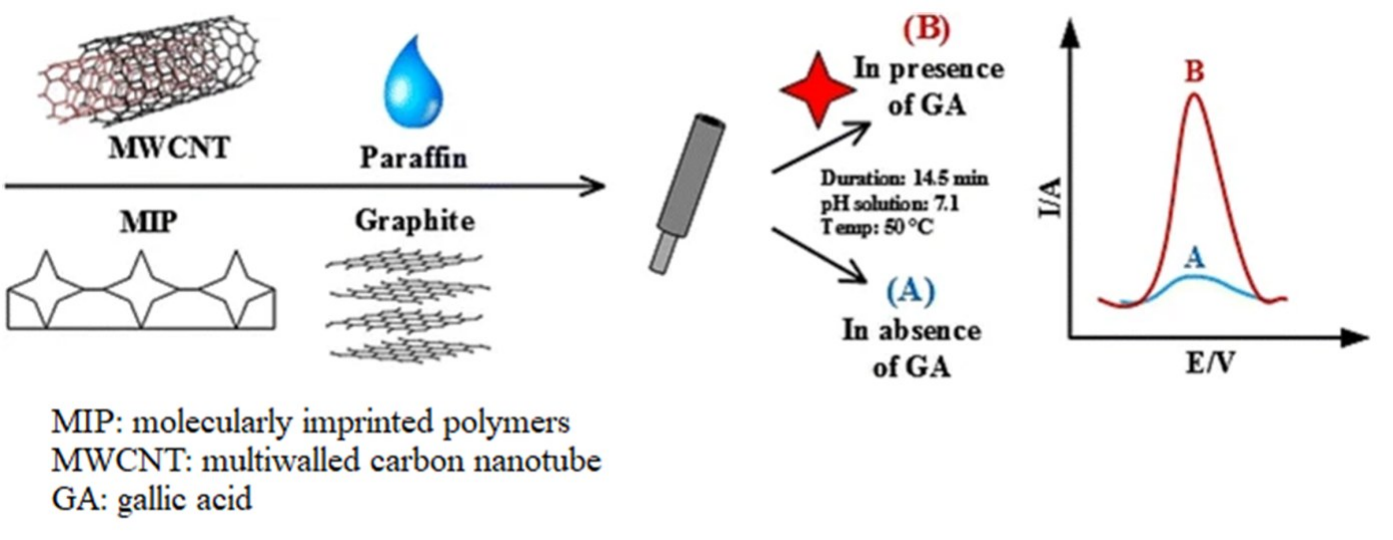
电化学纳米MIPs传感器的工作过程^［134］^

**表1:** MIPs传感器电化学检测分析的应用示例

Target	Sample	Molecularly imprinted polymers	LOD/（μmol/L）	Ref.
Folic acid	water	poly（*o*-phenylenediamine/*N*-isopropylacrylamide-*co*-*N，N*-methylbisacrylamide）	0.9	［^136^］
2，4，6-Tribromophenol	water	poly（*β*-cyclodextrin-*co*-vinyl butyraldehyde）	6.3×10^-4^	［^137^］
1，3-Dinitrobenzene	water	polyaniline	7.3×10^-3^	［^138^］
Kanamycin	milk	polypyrrole/graphene oxide composite	5.0×10^-3^	［^131^］
Caffeic acid	wine	poly（*N*-phenylpropanamide/methacrylic acid-*co*-ethylene glycol dimethacrylate）	130	［^139^］

### 3.3 电场作用下MIPs的再生

在检测分析后，MIPs传感器需经溶剂洗脱再生，以便于后续的循环利用。在外加电场作用下，电泳驱动力可辅助洗脱MIPs传感器上的目标物质，实现材料的再生，避免传统的洗脱方法可能存在洗脱不完全或对材料结构造成损伤的问题^［140］^。通过控制电场的强度和作用时间，可以实现高效、温和的洗脱过程，减少对材料的损害，提高MIPs传感器的使用寿命和重复使用性能。Wei等^［140］^在玻碳电极表面制备了以牛血清白蛋白为模板分子的MIP涂层，并用作电化学检测的传感器。该传感器在使用过后，可不使用解吸溶剂，仅通过在-0.8~0.8 V范围内电位循环14次，即可有效去除传感器上的目标物质，显示出良好的自清洁功能。

## 4 结语

电场辅助技术与MIT的融合为MIPs的发展提供了新契机。在制备过程中，外加电场可加速预聚合反应、调控印迹结构、有利于原位制备和表面修饰，为制备高特异性识别性能的MIPs提供了新途径；在样品前处理中，外加电场可加速吸附和解吸过程、提高MIPs的特异性识别能力、实现在线处理，提升了样品前处理的效率和质量；检测分析时，外加电场可增强传质和信号、助力洗脱再生，使检测更加灵敏和准确。今后，电场辅助MIT仍需大力发展。首先，要注重基础研究的深入，探索电场作用下MIPs的新性能和新应用。例如，研究外加电场对不同类型模板分子和功能单体预聚合的影响，开发更具特异性和适用性的MIPs。其次，应将电场辅助MIT拓展到离子印迹材料制备中，发展以重金属或无机阴离子为模板的特异性识别材料制备中。第三，要将电场辅助MIT与更多的萃取模式相结合，如管内固相微萃取技术，在改善萃取选择性的同时，将样品制备过程与分析检测技术在线结合，提高操作简便性和灵活性。第四，加强技术创新和应用转化，将电场辅助MIT推广到更多领域。如在环境监测中，实现对痕量污染物的实时在线监测；在生物医学领域，开发高灵敏度的生物传感器用于疾病诊断。本文通过对电场辅助MIT在不同应用层面的系统梳理与探讨，期望能够为该领域的科研工作者提供多维度的思考视角与切实可行的技术参考，助力推动该技术的发展。同时，也期待能够激发更多创新思路，促进该技术在环境监测、生物医学诊断、食品安全检测等领域的更广泛应用。
